# Changing Face of Inflammatory Activation in Complex Coronary Artery Disease during the COVID-19 Pandemic

**DOI:** 10.3390/jcdd10050199

**Published:** 2023-04-30

**Authors:** Tomasz Urbanowicz, Paweł Czub, Anna Olasińska-Wiśniewska, Michał Michalak, Zuzanna Fryska, Jakub Zieliński, Krzysztof Jerzy Filipiak, Krzysztof Wróbel, Andrzej Tykarski, Marek Jemielity

**Affiliations:** 1 Cardiac Surgery and Transplantology Department, Poznan University of Medical Sciences, 61-848 Poznan, Poland; 2 Cardiac Surgery Department, Lazarski University, 02-662 Warszawa, Poland; 3 Department of Computer Science and Statistics, Poznan University of Medical Sciences, 61-701 Poznan, Poland; 4 Faculty of Medicine, Poznan University of Medical Sciences, 61-701 Poznan, Poland; 5 Institute of Clinical Science, Maria Sklodowska-Curie Medical Academy, 00-001 Warsaw, Poland; 6 Department of Hypertensiology, Angiology and Internal Medicine, Poznan University of Medical Sciences, 61-848 Poznan, Poland

**Keywords:** COVID-19, coronary artery disease, inflammatory activation, mortality, MLR, SIRI, NLR

## Abstract

Introduction: The COVID-19 pandemic has changed the immunological status of the population, indicating increased activation. The aim of the study was to compare the degree of inflammatory activation in patients admitted for surgical revascularization in the period before and during the COVID-19 pandemic. Materials and methods: This retrospective analysis included an analysis of inflammatory activation assessed on the basis of whole blood counts in 533 patients (435 (82%) male and 98 (18%) female) with a median age of 66 (61–71) years who underwent surgical revascularization, including 343 and 190 patients operated on in 2018 and 2022, respectively. Results: The compared groups were matched by propensity score matching analysis, obtaining 190 patients in each group. Significantly higher values of preoperative monocyte count (*p* = 0.015), monocyte-to-lymphocyte ratio (*p* = 0.004) and systemic inflammatory response index (*p* = 0.022) were found in the during-COVID subgroup. The perioperative and 12-month mortality rates were comparable, with 1% (*n* = 4) in 2018 vs. 1% (*n* = 2) in 2022 (*p* = 0.911), and 5.6 % (*n* = 11 patients) vs. 7% (*n* = 13 patients) (*p* = 0.413), in the pre-COVID and during-COVID subgroups, respectively. Conclusions: Simple whole blood analysis in patients with complex coronary artery disease performed before and during the COVID-19 pandemic indicates excessive inflammatory activation. However, the immune variation did not interfere with one-year mortality rate after surgical revascularization.

## 1. Introduction

Fewer hospitalizations, followed by a lower number of performed procedures, were reported during the coronavirus disease 2019 (COVID-19) pandemic [1]. The number of surgical revascularization procedures has decreased due to the reorganization of the healthcare system, although the overall number of percutaneous procedures has not been reduced [2]. The time from the onset of pain to the first medical contact significantly extended during the lockdown, as did the number of hospitalizations due to acute coronary syndromes [3].

A decrease in the overall total number of cardiac surgical operations, including coronary artery bypass grafting, was reported [4]. The COVID-19 pandemic has also changed the immunological status of the population, indicating increased activation, e.g., higher levels of monocyte chemoattractant chemokines protein–1 (MCP-1) or interferon gamma- induced protein 10 (IP-10), with no association with clinical status [5]. The neuropsychiatric disorders manifested by cognitive and mood disorders secondary to immune activation after COVID-19 infection were presented by Bove et al. [6]. The reported infection rate in the population was as high as 50% [7], with the seroprevalence of severe acute respiratory syndrome coronavirus-2 (SARS-CoV-2) antibodies in the general population estimated at 13% [8]. A mortality prediction algorithm for COVID-19 pneumonia was proposed [9], while, importantly, up to 40% patients may be asymptomatic [10].

The SARS-CoV-2 pandemic had a significant impact on immune status in certain groups of patients, which may result in a specific immune cell response in the form of delayed-type hypersensitivity [11]. A possible increase in diseases with an inflammatory background has been reported in the COVID-19 era [12,13], suggesting an immunologically triggered etiology. Post-COVID-19 syndrome manifestations include immunological disturbances related to molecular and cellular immune abnormalities caused by the host’s initial violent immune response, including cytokine release syndrome, hemophagocytic lymph histiocytosis, and macrophage activation syndrome [14]. The post-vaccination exacerbations in autoimmune inflammatory diseases were presented in a study by Ma et al. [15].

The inflammatory background of coronary artery disease has been postulated [16,17], indicating its significance in the etiology and progression of the disease. The inflammatory activation, measured by peripheral neutrophil-to-lymphocyte ratio (NLR), was found to be a prognostic factor of the severity of coronary lesions and mortality in acute coronary syndromes [18]. Inflammatory indexes obtained from peripheral whole blood count analysis have gained much attention in recent years due to their availability and cost-effectiveness. Their role as flags of immune system disorders has been established.

Inflammatory activation, assessed using peripheral blood indexes based on counts of monocytes, neutrophils, lymphocytes, and platelets, has been considered as predictive in oncology [19,20], transplantology [21], and metabolic disorders [22,23]. Recent publications have shown relationship between NLR and risk of admission to the intensive care units among patients with COVID-19 [24].

Among inflammatory indexes, the significance of NLR for long-term prognosis has been revealed in percutaneous interventions [25] and surgical revascularization [26]. In our previous study, a perioperative inflammatory reaction measured by NLR was proposed, together with clinical and echocardiographical parameters, as a part of a long-term mortality risk-score [27]. The novel approach seems to be justified, since standard validators of long-term prognosis in cardiac surgery are characterized by limited discriminatory power [28]. 

The correlation between expression of serum level of inflammatory cytokines and markers of oxidative stress, and coronary artery calcium score was presented by Yao et al. [29].

The activation of monocytes is claimed to have an influence on the evolution of atherosclerotic plaques’ [30,31]. Gospodarczyk et al., in their review [32], demonstrated the role of the inflammatory response in the development of atherosclerosis in patients with viral SARS-CoV-2 infection. The inflammatory activation possesses prognostic significance for long-term survival in patients with diagnosed complex coronary artery disease [33,34]. 

The aim of the study was to compare the degree of inflammatory activation between patients admitted for surgical revascularization in the period before and during the COVID-19 pandemic.

## 2. Materials and Methods

There were 533 patients (435 (82%) male and 98 (18%) female) with a median age of 66 (61–71) years referred for surgical revascularization in two cardiac surgery centers from different geographical regions in Poland. Consecutive patients referred for elective surgery were enrolled into the study. The retrospective analysis included analysis of inflammatory activation calculated from the whole blood count results of 343 patients operated in 2018 and 190 patients in 2022. Patients with a history of acute coronary syndromes (unstable angina or acute myocardial infarction), acute myocarditis, acute stroke, inflammatory diseases, and oncological and hematological diseases were excluded from the study. Chronic obstructive pulmonary disease (COPD) was defined according to the GOLD criteria; peripheral artery disease (PAD) was classified as at least 50% stenosis in peripheral artery; diagnosis of stroke in history was based on the results of computed tomography/magnetic resonance imaging; nicotinism was defined as active smoking and kidney disease was defined by glomerular filtration rate (GFR) being lower than 60 ml/min/1.73m2. Detailed characteristics are presented in Table 1.

### Statistical Analysis

Data were tested for normality with the Shapiro–Wilk test. Continuous data were not normally distributed and were presented as medians and interquartile range (Q1–Q3), and compared using the nonparametric Mann–Whitney test. Categorical variables were presented as counts and percentages and compared with the chi-square test. Kaplan–Meier curves were performed to estimate survival. The log-rank test was applied to compare survival between the analyzed groups. A logistic regression was performed to find factors which predict mortality risk. Both univariate and multivariate analyses were performed. The multivariate logistic regression model with backward stepwise elimination method was denoted and the results were presented as odds ratio and its 95% confidence interval. Statistical analysis was performed using MedCalc^®^ Statistical Software version 20.027 (MedCalc/Software Ltd., Ostend, Belgium). Values of *p* < 0.05 were considered statistically significant.

## 3. Results

**Figure 1:**
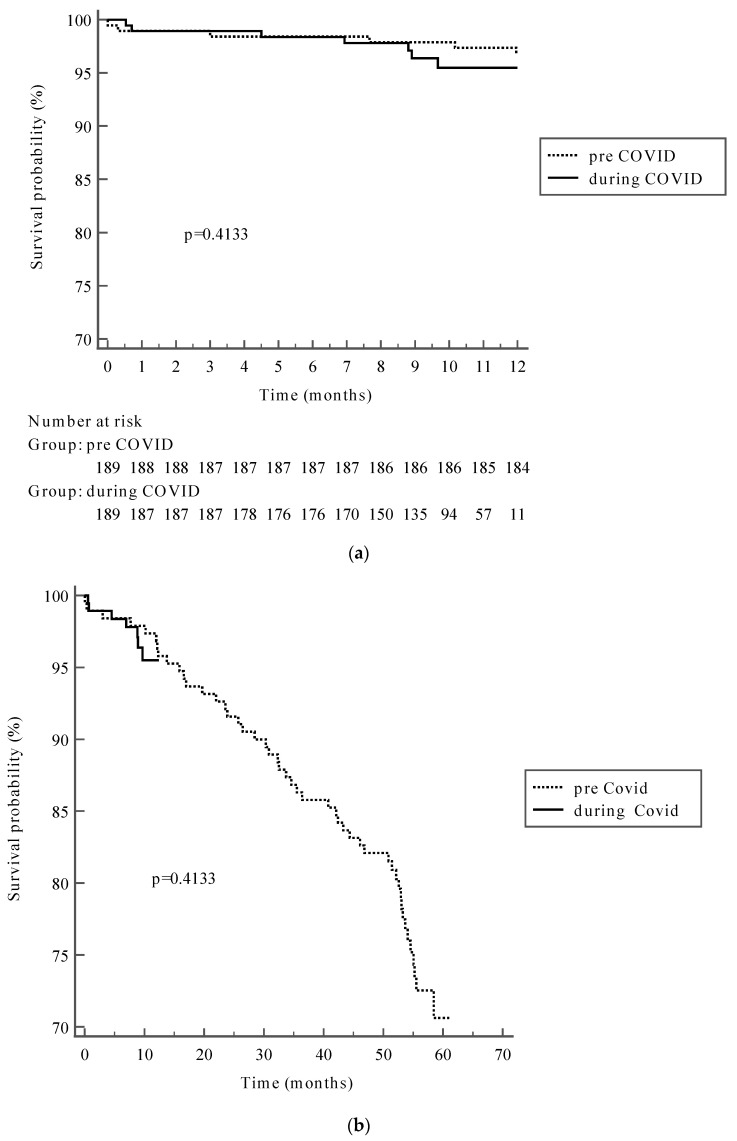
(**a**) One-year survival in pre-COVID and during-COVID groups. (**b**) Long-term survival in pre-COVID and during-COVID groups. The 4-year survival rate for the whole group was 86% (458 patients). Among the patients operated on in 2022, there were 161 (85%) who were vaccinated with at least two standard doses against COVID-19 with median 260 (211–301) days prior to the surgery.

The perioperative mortality rate in the presented study was 1% (*n* = 6 patients) including 1% (*n* = 4) of patients operated on in 2018 vs. 1% (*n* = 2) in 2022 (*p* = 0.911). The 12-month mortality was reported as 5.6% (*n* = 11 patients) and 7% (*n* = 13 patients) in the log-rank test results (*p* = 0.413), in pre-COVID 19 and during-COVID periods, respectively, as presented in Figure 1a,b.

The median hospitalization time in the studied periods was 12 (9–15) days vs. 11 (9–14) days (*p* = 0.798), respectively. The median number of performed anastomoses in each group was 2 (2–3) vs. 2 (2–3) (*p* = 0.950), respectively.

The compared groups were matched by propensity score matching analysis, obtaining 190 patients in each group. The demographic and clinical characteristics of both matched groups did not reach any statistical significance, as presented in Table S1. Preoperative laboratory results between both matched groups did not reach any significance regarding kidney function (*p* = 0.978) or liver function tests (*p* = 0.644 and *p* = 0.856, respectively). Although the preoperative myocardial injury markers were within normal limits, statistical significance was observed (*p* = 0.007). As previously mentioned, the analysis of myocardial injury and inflammatory markers, together with clinical status, excluded those patients with acute coronary syndromes and acute myocarditis. Preoperative laboratory results of patients referred for surgical revascularization are presented in Table 2.

Thereafter, the uni- and multivariable analysis for 12 months mortality risk pre-diction for both evaluated groups was performed, as presented in Table 3.

The uni- and multivariable analysis for 12-month mortality risk prediction for separate groups was performed and presented in Table S1 (Supplementary Materials).

## 4. Discussion

The main finding of our study is related to the significant difference in preoperative monocyte counts and systemic inflammatory response index (SIRI) results between patients with complex coronary artery disease treated before and during the COVID-19 pandemic. The propensity matching of two groups of patients operated on before and during the COVID-19 pandemic was performed to obtain comparable populations. Our analysis showed differences in inflammatory activation in patients in the during-COVID group, since monocyte count, MLR, SIRI, and AISI were all at higher values than in the pre-COVID group. 

To our knowledge, this is the first such observation in the literature which may provide an explanation for COVID-19 consequences, long COVID syndromes, and the intriguing reports of increased mortality in any type of operation performed within 7 weeks after SARS-CoV-2 infection [35].

This phenomenon was not significant for one-year mortality, but as mentioned earlier, the time perspective of such inflammatory response in the during-COVID group was limited to several weeks. Fesu et al. presented functional impairment, presenting as decreased walking distance and desaturation, within 10 weeks following SARS-CoV-2 infection [36]. Neurological complications persisting 3 months after the acute phase of viral infection were underlined by Ariza et al. [37].

In our previous analysis, SIRI was shown as a prognostic marker for long-term results in patients undergoing surgical revascularization [15]. Further studies are required to determine its influence in patients who undergo surgical revascularization due to complex coronary artery disease in different settings. Our results present 1-year mortality after surgical revascularization during the COVID-19 pandemic, which are comparable to the pre-COVID population. We believe that long-term observation is required to draw objective conclusions, as the main advantage of surgical revascularization is related to long-term results [38]. As postulated in Jonik et al.’s study, the multidisciplinary team decision on the therapeutic approach is based on the general health status of patients referred for intervention [39].

Inflammatory markers obtained from the peripheral blood analysis have been found to possess significant value for the prediction of complications in a broad spectrum of diseases [40,41,42]. The indexes are composed of neutrophil, monocyte, lymphocyte, and platelet counts, and can be incorporated into analysis of innate and adaptive immunological response [43]. Neutrophils regulate processes such as acute injury and repair, and contribute to adaptive immunity [44]. Monocytes are claimed to contribute to the pathogenesis of inflammatory reactions [45]. Lymphocytes are responsible for adaptive immunity [46]. The role of platelets, another component of inflammatory indexes, is related to chemokine activation, which may lead to immune-mediated cardiovascular complications [47]. 

As shown in Tan et al.’s study, in COVID-19 pneumonia, lymphocyte count decrease correlated with disease severity [48]. The most notable decrease was observed within NK and CD8+ T cells, and their function was also impaired as there was an increased expression of the inhibitory receptors noted [49]. In Zheng et al.’s study, the functional exhaustion of cytotoxic lymphocytes in COVID-19 patients was found and claimed to be associated with the acute phase of infection [50]. Importantly, Antonioli et al. [51] revealed an impairment in the interferon alpha (IFN-γ) defense mechanism in COVID-19 patients, which allowed for accumulation of pathogenic neutrophils. 

Cata et al. [52], in their study, presented a higher rate of 30-day neurological complications in cancer surgery compared with pre-pandemic non-COVID-19 patients, though postoperative use of antipsychotic medications was not significantly different.

Our multivariable analysis presented age, preoperative white blood cell count (WBC), and red blood cell distribution width (RDW) as significant predictive factors for one-year mortality. These parameters did not differ between matched groups. Therefore, although our analysis and previous reports [53,54] showed a particular change in immune activation during the COVID-19 pandemic, this did not seem to have a significant impact on one-year mortality after cardiac surgery. 

The systemic inflammatory response index describes neutrophil and monocyte count increase in peripheral blood compared to lymphocyte count decline. Our results indicate that monocyte count in the whole blood count analysis varied after the COVID-19 pandemic in a retrospective analysis of two subgroups of patients from different regions of our country. This observation may indicate the common characteristics which may play a prognostic role in patients suffering from coronary atherosclerosis. Monocyte activity is a part of innate and adaptive response to different pathogens, including viral infections. Hopkins et al. analysis [55] revealed a persistent elevation of monocytes levels following SARS-CoV-2 infection, attributed to classical monocytes. Additionally, Toribio et al. [56] presented a correlation between myocardial performance and peripheral blood monocytes’ activation in active viral infection. The comprehensive response of immunological system, secondary to vaccination, includes monocytes and antigen presentation T cells’ activation. Both pathways’ upregulation in cell–cell enhanced communications between innate and adaptive immunity was reported by Liu et al. [57]. The relation between chronic inflammatory diseases and monocyte subtypes has been presented by Kapellos et al. [58]. Animal studies revealed that circulating monocytes play a pivotal role in inflammatory activation during SARS-CoV-2 infection [59]. Prolonged monocyte activation following viral infection has been related to their anti-apoptotic functions [60,61]. The relation between circulating monocyte subtypes and cardiovascular risk prediction was presented in a review by Ożańska et al. [62]. 

Several authors presented the predictive value of inflammatory indexes, including neutrophil-to-lymphocyte ratio (NLR), evaluated during the acute phase of SARS-CoV-2 infection, with significance for disease severity [63], mechanical ventilation [64], and mortality [65].

Monocyte-to-lymphocyte ratio was postulated as representative of blood-count-derived inflammatory markers in females for prediction of cardiovascular risk [66]. Xiang et al., in their observational study, presented the monocyte upregulation secondary to low serum concentration of high-density lipoprotein cholesterol (HDL-C) in coronary artery disease patients [67]. Non-classical monocyte subtypes (CD14+, CD16+) were postulated as independent predictors of cardiovascular events in patients referred for elective coronary angiography [68].

Beside voluminous reports of the role of monocytes in atherosclerosis progression, the significance of SIRI in cardiovascular outcomes was presented in previous studies, including cerebrovascular syndromes [69], heart failure [70], and acute/chronic coronary syndromes [71,72]. The SIRI index after adjustment for age and creatinine level was postulated by Yildiz et al. as an independent predictor of one-year major adverse cardiovascular events [73]. In a cohort study with 20-year follow-up, SIRI was explored as a novel prognostic marker for cardiovascular and all-cause mortality [74]. SIRI is also claimed to be a novel and convenient measurement in patients with ischemic stroke that positively associates with atrial fibrillation, an arrhythmia which is also commonly related to inflammatory activation [75]. 

In the presented results, we found a significant difference in the diagnosis of peripheral artery disease between pre-COVID and during the COVID-19 pandemic. This may be explained by worse accessibility to diagnostic procedures and therefore lower possibilities for proper vascular disease diagnosis due to pandemic restrictions. Kasiri et al. [76] presented an increased number of emergency admissions with unsuccessful revascularization attempts requiring limb amputation, due to pandemic lockdown protocols which limited ability for accurate planned diagnosis.

The presented studies did not show a significant difference in mortality due to cardiovascular disease comparing the time before the pandemic and the COVID-19 pandemic, though significant differences between countries were postulated [77]. Increased mortality was related to lockdown restrictions rather than to the change of performed procedures [78]. Our study is the first, to the best of our knowledge, to present the possible relationship between COVID-19-pandemic-related immunological activation in the human population and the possible impact on long-term prognosis. The presented optimal results, despite the increased inflammatory activation prior to surgery [79], may be explained by accurate pharmacotherapy, including statins, in the presented group, as in Azimi’s study [80]. Katsiki et al. [81], in their study, presented the beneficial effects of lipid-lowering therapy in COVID-19, e.g., by proteases [82].

Complications of SARS-CoV-2 infection, such as long COVID syndrome, relate to abnormalities reported among patients beyond the acute phase and include myocardial inflammation, dysfunction, infarction, or arrhythmias [83]. Pathophysiological mechanisms are still poorly understood, but may be also associated with autonomic dysfunction, as presented by Dani et al. [84]. Castanares-Zapatero et al. [85], in their review, suggested that specific long-lasting inflammatory mechanisms are the underlying mechanism. Neurological complications following SARS-CoV-2 infection were hypothesized to be related to combined sustained neuroinflammation [86] and local microthrombosis [87]. Despite our 1-year non-inferior results in patients undergoing surgical revascularization in the COVID-19 pandemic, the observation of elevated levels of inflammatory markers may anticipate the risk of future complications, which indicates scrupulous monitoring of these patients. 

Study limitations:

This is a retrospective study presenting differences in laboratory results between the pre- and during-COVID-19 pandemic periods. Though we have anticipated worse long-term results in patients during the COVID-19 pandemic undergoing cardiovascular procedures based on preoperative results and previous publications [88,89], we believe that the verification of the presented thesis requires a longer follow-up. In the presented results, we found differences in peripheral artery disease diagnosis between patients before and during the COVID-19 pandemic that may point out the possible logistic and medical side-effects of the pandemic. Our analysis was based on inflammatory indexes obtained from whole blood count analysis. We did not include C-reactive protein (CRP) serum concentration, as this parameter was measured only on suspicion of perioperative infection and not as a routine measurement.

## 5. Conclusions

Simple whole blood analysis in patients with complex coronary artery disease performed before and during the COVID-19 pandemic indicated excessive inflammatory activation. The immune change did not, however, interfere with one-year mortality rate after coronary artery bypass grafting.

## Figures and Tables

**Table 1:** Preoperative demographic and clinical characteristics of patients referred for surgical revascularization.

Parameters	Pre-COVID	During COVID	P
	2018	2022	
	*n* = 190	*n* = 190	
Demographical characteristics			
Sex (M (%)/F (%))	160 (84)/30 (16)	158 (83)/32 (17)	0.781
Age (median (Q1–Q3) years	67 (62–72)	67 (63–72)	1
Weight (median (Q1–Q3) kg	80 (70–89)	80 (72–89)	0.986
Height (median (Q1–Q3) cm	170 (164–176)	171 (167–176)	0.273
BMI (median (Q1–Q3)	28 (25–30)	28 (25–30)	0.468
Co-morbidities:			
Arterial hypertension (*n* (%))	151 (79)	145 (76)	0.458
Hypercholesterolemia (*n* (%))	99 (52)	96 (51)	0.758
DM (*n* (%))	69 (37)	66 (35)	0.69
DM insulin-dependent (*n* (%))	7 (4)	13 (7)	0.168
COPD (*n* (%))	12 (6)	13 (7)	0.836
PAD (*n* (%))	37 (19)	23 (12)	0.049
Stroke (*n* (%))	6 (3)	2 (2)	0.311
Kidney failure (*n* (%))	5 (3)	8 (4)	0.397
Nicotinism (*n* (%))	50 (26)	55 (52)	0.566

Abbreviations: BMI—body mass index, COPD—chronic obstructive pulmonary disease, DM—diabetes mellitus, F—female, M—male, PAD—peripheral artery disease, Q—quartile.

**Table 2:** Preoperative laboratory results of patients referred for surgical revascularization.

Parameters	Pre-COVID	During COVID	P
	2018	2022	
	*n* = 190	*n* = 190	
Whole blood count analysis:			
WBC (K/uL) (median (Q1–Q3) Lymphocytes (K/uL) (median (Q1–Q3) Neutrophils (K/uL) (median (Q1–Q3) Monocytes (K/uL) (median (Q1–Q3) Hemoglobin (mmol/L) (median (Q1–Q3) Hematocrit (%) (median (Q1–Q3) Platelets (K/uL) (median (Q1–Q3) NLR MLR SII SIRI AISI MPV (fl) (median (Q1–Q3) RDW (%) (median (Q1–Q3)	7.68 (6.43–9.17)	7.64 (6.42–8.88)	0.283
	1.84 (1.46–2.36)	1.79 (1.44–2.16)	0.113
	4.81 (3.99–5.95)	4.91 (3.88–5.76)	0.862
	0.49 (0.37–0.62)	0.54 (0.41–0.69)	0.015 *
	10.15 (8.4–12.1)	11.95 (9.0–13.9)	0.061
	40.8 (37.7–43)	38.7 (36.2–40.6)	0.057
	217 (189–265)	229 (195–268)	0.225
	2.59 (1.95–3.55)	2.81 (2.0–3.56)	0.373
	0.27 (0.19–0.38)	0.30 (0.24–0.38)	0.004 *
	>587 (405–819)	636 (460–874)	0.197
	1.26 (0.85–1.83)	1.43 (1.03–2.08)	0.022 *
	281 (177–434)	325 (220–540)	0.024 *
	8.75 (7.9–10.5)	9.7 (8.8–10.5)	0.000 *
	13.3 (12.9–14)	13.2 (12.7–13.8)	0.155
Myocardial injury marker:			
Troponin-I (ng/L) (median (Q1–Q3)	0.004 (0.000–0.030)	0.010 (0.003–0.031)	0.007 *
Kidney function tests:			
Serum creatinine mmol/L (median (Q1–Q3))	91 (76–103)	91 (74–104)	0.978
Liver function test:			
ALT IU/dL (median (Q1–Q3)	30 (22–42)	29 (21–39)	0.644
2. AST IU/dL (median (Q1–Q3)	27 (21–36)	28 (21–38)	0.856
Lipid profile:			
Total cholesterol (mmol/L) (median (Q1–Q3)	3.50 (3.07–4.24)	3.51 (3.08–4.27)	0.762
2. HDL fraction (mmol/L) (median (Q1–Q3)	1.10 (0.92–1.29)	1.10 (0.89–1.29)	0.803
3. LDL fraction (mmol/L) (median (Q1–Q3)	2.00 (1.63–2.53)	1.98 (1.53–2.51)	0.772

Abbreviations: AISI—aggregate index of systemic inflammation, ALT—alanine aminotransferase, AST—aspartate aminotransferase, GFR—glomerular filtration rate, HDL—high-density lipoprotein cholesterol, LDL—low-density lipoprotein cholesterol, MLR—monocyte-to-lymphocyte ratio, MPV—mean platelet volume, NLR—neutrophil-to-lymphocyte ratio, RDW—red cell distribution width, SII—systemic inflammatory index, SIRI—systemic inflammatory response index, WBC—white blood cell count, * statistically significant.

**Table 3:** Uni- and multivariable analysis for 12-month mortality prediction in both groups (pre-COVID and during-COVID group).

Parameters	Univariable Analysis			Multivariable Analysis		
	HR	95% CI	*p*-Value	HR	95% CI	*p*-Value
Demographical:						
Sex	0.84	0.44–1.61	0.598	-	-	-
Age	1.02	0.99–1.06	0.184	1.07	1.00–1.14	0.038 *
BMI	1.03	0.95–1.11	0.463	-	-	-
Clinical:					-	-
HA	0.88	0.48–1.61	0.684	-	-	-
DM	1.61	0.93–2.81	0.88	-	-	-
COPD	0.39	0.95–1.61	0.197	-	-	-
PAD	1.34	0.69–2.61	0.386	-	-	-
Stroke	1.88	0.59–6.05	0.288	-	-	-
Kidney failure	1.97	0.71–5.48	0.192	-		
Preoperative						-
LV ejection fraction	0.98	0.95–1.01	0.107	-	-	
Laboratory (whole blood count):						
WBC	1.05	0.99–1.11	0.081	1.23	1.00–1.34	0.038 *
Lymphocytes	1.03	0.95–1.13	0.483	-	-	-
Neutrophils	1.14	1.01–1.29	0.033 *	-	-	-
Monocytes	1.1	0.48–2.54	0.822	-	-	-
Hemoglobin	0.95	0.86–1.04	0.286	-	-	-
Hematocrit	0.97	0.94–1.01	0.096	-	-	-
Platelets	1	0.99–1.01	0.255	-	-	-
NLR	1.09	0.98–1.20	0.1	-	-	-
MLR	1.87	0.78–4.51	0.159	-	-	-
SII	1	0.99–1.01	0.053	-	-	-
SIRI	1.06	1.00–1.12	0.045 *	-	-	-
AISI	1	1.00–1.00	0.041	-	-	-
MPV	0.98	0.83–1.19	0.808	-	-	-
RDW	0.81	0.56–1.16	0.25	0.66	0.48–0.90	0.009 *
Laboratory (myocardial markers):						
Troponin I	1.04	0.98–1.1	0.222	-	-	-
Laboratory (liver function tests):						
AST	0.99	0.98–1.01	0.465	-	-	-
ALT	1	0.99–1.00	0.793	-	-	-
Laboratory (lipid profile):						
Total cholesterol	0.84	0.62–1.14	0.263	-	-	-
HDL	0.85	0.43–1.69	0.645	-	-	-
LDL	0.96	0.72–1.28	0.79	-	-	-
Number of performed grafts:	1.13	0.79–1.63	0.495	-	-	-

Abbreviations: AISI—aggregate index of systemic inflammation, ALT—alanine aminotransferase, AST—aspartate aminotransferase, BMI—body mass index, CI—confidence interval, COPD—chronic obstructive pulmonary disease, DM—diabetes mellitus, HA—arterial hypertension, HDL—high-density lipoprotein cholesterol, HR—hazard ratio, LDL—low-density lipoprotein cholesterol, MLR—monocyte-to-lymphocyte ratio, MPV—mean platelets volume, NLR—neutrophil-to-lymphocyte ratio, PAD—peripheral artery disease, RDW—red cell distribution width, SII—systemic inflammatory index, SIRI—systemic inflammatory response index, * statistically significant.

## Data Availability

The data are available upon reasonable request to the corresponding authors within 3 years following publication (turbanowicz@ump.edu.pl).

## Supplementary Materials

The following supporting information can be downloaded at: https://www.mdpi.com/article/10.3390/jcdd10050199/s1, Table S1: Uni- and multivariable analysis for 12-month mortality prediction in pre-COVID and post-COVID group (performed separately).
