# Supplementary material for: Changing Face of Inflammatory Activation in Complex Coronary Artery Disease during the COVID-19 Pandemic
Source: J Cardiovasc Dev Dis. 2023 Apr 30;10(5):199. doi: 10.3390/jcdd10050199 (PMC10219314; doi:10.3390/jcdd10050199)
Supplement: Supplementary file 1 [file jcdd-10-00199-s001.zip › jcdd-2331382-supplementary.pdf]

Supplementary Materials

Table S1. Uni- and multivariable analysis for 12-month mortality prediction in pre-COVID and post-COVID group (performed separately).

Parameters	Pre- COVID						Post-COVID					
	Univariable Analysis			Multivariable Analysis			Univariable Analysis			Multivariable Analysis		
	HR	95% CI	p - value	HR	95% CI	p - value	HR	95% CI	p - value	HR	95% CI	p - value
Demographical:												
Sex	0.95	0.42 – 2.07	0.849	-	-	-	0.51	0.09 – 2.62	0.418	-	-	-
Age	1.04	0.99 – 1.08	0.052	-	-	-	1.04	0.93 – 1.16	0.516	-	-	-
BMI	1.08	0.99 – 1.17	0.066	-	-	-	1.14	0.94 – 1.39	0.190	-	-	-
Clinical:												
HA	1.17	0.57 – 2.42	0.670	-	-	-	1.97	0.24 – 16.36	0.531	-	-	-
DM	1.34	0.82 – 5.28	0.337	-	-	-	0.71	0.14 – 3.69	0.687	-	-	-
COPD	2.08	0.95 – 1.61	0.121	-	-	-	2.26	0.27 – 18.83	0.449	-	-	-
PAD	1.73	0.91 – 3.28	0.093	-	-	-	1.22	0.15 – 10.18	0.851	-	-	-
Stroke	3.20	0.99 – 10.31	0.051	-	-	-	1.66	0	1.00	-	-	-
Kidney failure	0.74	0.10 - 5.35	0.763	-	-	-	3.26	0.39 – 27.14	0.274	-	-	-
Hypercholesterolemia	1.07	0.60 – 1.89	0.819				1.24	0.28 – 5.55	0.777			
Preoperative LV ejection fraction	0.98	0.95 – 1.01	0.148	-	-	-	0.96	0.91 – 1.02	0.211	-	-	-
Laboratory (Whole blood count):												
WBC	1.09	1.04 – 1.15	<0.001	-	-	-	1.04	0.72 – 1.50	0.826	-	-	-
Lymphocytes	1.10	1.04 – 1.17	0.002	1.1	1.03 – 1.17	0.002	1.19	0.46 – 3.09	0.714	-	-	-
Neutrophils	1.13	1.00 – 1.28	0.049	-	-	-	1.07	0.68 – 1.67	0.777	-	-	-
Monocytes	1.01	0.50 – 2.03	0.980	-	-	-	0.03	0.00 – 4.51	0.166	-	-	-
Hemoglobin	0.89	0.79 – 1.02	0.100	-	-	-	0.97	0.73 – 1.29	0.823	-	-	-
Hematocrit	0.89	0.79 – 1.02	0.093	-	-	-	0.99	0.96 – 1.03	0.750	-	-	-

Platelets	1.00	0.99 – 1.00	0.889	-	-	-	1.01	0.99 – 1.01	0.112	-	-	-
NLR	1.04	0.92 – 1.17	0.552	-	-	-	0.89	0.49 – 1.61	0.715	-	-	-
MLR	0.926	0.33 – 2.58	0.883	-	-	-	0.00	0.00 – 1.93	0.068	-	-	-
SII	1.00	0.99 – 1.01	0.295	-	-	-	1.00	0.99 – 1.00	0.496	-	-	-
SIRI	1.04	0.98 – 1.12	0.155	-	-	-	0.52	0.15 – 1.79	0.297	-	-	-
AISI	1.00	1.00 – 1.00	0.102	-	-	-	1.00	1.00 – 1.00	0.876	-	-	-
MPV	0.97	0.81 – 1.17	0.780	-	-	-	1.05	0.59 – 1.85	0.871	-	-	-
RDW	0.75	0.55 – 1.01	0.062	-	-	-	1.02	0.46 – 2.26	0.970	-	-	-
Laboratory (myocardial markers):												
Troponin I	0.59	0.07 – 5.41	0.644	-	-	-	0.46	0.00 – 436.73	0.824	-	-	-
Laboratory (liver function tests):												
AST	0.99	0.97 – 1.00	0.084	-	-	-	0.99	0.96 – 1.03	0.718	-	-	-
ALT	0.99	0.99 – 1.01	0.738	-	-	-	1.02	0.96 – 1.07	0.562	-	-	-
Laboratory (lipid profile):												
Total cholesterol	0.79	0.56 – 1.11	0.171	-	-	-	0.93	0.48 – 1.79	0.816	-	-	-
HDL	0.73	0.32 – 1.66	0.454	-	-	-	0.13	0.01 – 2.32	0.166	-	-	-
LDL	0.95	0.69 – 1.31	0.758	-	-	-	0.93	0.41 – 2.10	0.857	-	-	-
No of performed grafts:	0.99	0.66 – 1.49	0.982	-	-	-	1.09	0.47 – 2.52	0.847	-	-	-

Abbreviations: AISI – aggregate index of systemic inflammation, ALT – alanine aminotransferase, AST – aspartate aminotransferase, BMI – body mass index, CI – confidence interval, COPD – chronic obstructive pulmonary disease, DM – diabetes mellitus, HA- arterial hypertension, HDL – high density lipoprotein cholesterol, HR – hazard ratio, LDL – low density lipoprotein cholesterol, LV- left ventricular, MLR – monocyte to lymphocyte ratio, MPV – mean platelets volume, NLR – neutrophil to lymphocyte ratio, PAD – peripheral artery disease, RDW – red cells distribution width, SII – systemic inflammatory index, SIRI – systemic inflammatory response index, WBC – white blood cells, * statistically significant.
